# Scorpionfish adjust skin pattern contrast on different backgrounds

**DOI:** 10.1002/ece3.11124

**Published:** 2024-03-11

**Authors:** Leonie John, Matteo Santon, Nico K. Michiels

**Affiliations:** ^1^ Animal Evolutionary Ecology Institute of Evolution and Ecology, University of Tübingen Tübingen Germany; ^2^ Ecology of Vision Group, School of Biological Sciences University of Bristol Bristol UK

**Keywords:** background matching, camouflage, colour change, disruptive colouration, pattern energy analysis, QCPA

## Abstract

The two scorpionfish species *Scorpaena maderensis* and *S. porcus* are well camouflaged ambush predators that rapidly change body colouration to adjust to background colour in less than 1 min. We tested whether individuals of both species also adjust body pattern to that of the background. We placed fish on backgrounds of different pattern granularity and quantified the change in fish body pattern over 1 min. We used calibrated image analysis to analyse the patterns from the visual perspective of a prey fish species using a granularity (pattern energy) analysis and an image clustering approach. In our experiment, fish did not change their most contrasting pattern components as defined by the dominant marking size, but changed their average marking size. Moreover, fish responded with a change in pattern in contrast to the different experimental backgrounds, especially when compared to the acclimation phase. These results indicate that scorpionfish have one main pattern that can be adjusted by modulating its internal contrast. A reduction in pattern contrast could thereby improve background matching, while an increase could promote camouflage via disruptive colouration.

## Graphical abstract

Scorpionfishes are well‐camouflaged ambush predators. We tested whether they changed their skin pattern in response to background pattern from the visual perspective of prey. Our data shows that fish adjust internal pattern contrast to adapt to patterns of different granularity, and we discuss how dynamic change in pattern can contribute to camouflage via background matching or disruption.

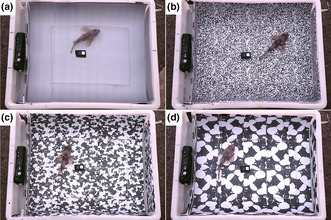

## 1 INTRODUCTION

Many animals use camouflage to hide from predators or prey, which can be achieved with different strategies (Stevens & Merilaita, 2009a). A body colouration and pattern very similar to that of the background could allow camouflage through background matching (Stevens & Merilaita, 2009a). Such a specific phenotype can, however, bring the disadvantage that camouflage is restricted to a specific background, often a background that is homogeneous in colouration and pattern (Briolat et al., 2021; Price et al., 2019). Disruptive colouration, on the other hand, can work on more heterogeneous backgrounds (Cuthill et al., 2005; Price et al., 2019; Robledo‐Ospina et al., 2017). Here, contrasting markings can create false edges and disrupt the body outline and shape, which makes it more difficult to be detected or recognised as such (Stevens & Merilaita, 2009b). Some animals dynamically change their body colouration and pattern, which can allow them to camouflage on multiple backgrounds, and to switch between camouflage strategies (Duarte et al., 2017).

Cuttlefish are renowned for their ability to change pattern in response to different backgrounds (Barbosa et al., 2008; Hanlon & Messenger, 1988; How & Santon, 2022; Mäthger et al., 2007; Osorio et al., 2022). Highly variable patterns can be produced by the high‐dimensional control and flexible grouping of chromatophores (Woo et al., 2023). While cephalopods are unrivalled, fishes show remarkable pattern change too. Some flatfishes can switch between two to three different body patterns (Kelman et al., 2006; Ramachandran et al., 1996; Tyrie et al., 2015), and express up to six pattern components (Ramachandran et al., 1996). Nassau groupers and slender filefish display up to three body patterns in response to different natural substrates (Allen et al., 2015; Watson et al., 2014). The rock pool goby *Gobius paganellus* changes its pattern depending on the substrate granularity by modulating the contrast of certain bars within its pattern (Smithers et al., 2017). Many more fish species could potentially adjust body pattern for camouflage, as the ability to rapidly change colour is widespread in fishes (Nilsson Sköld et al., 2013).

**FIGURE 1:**
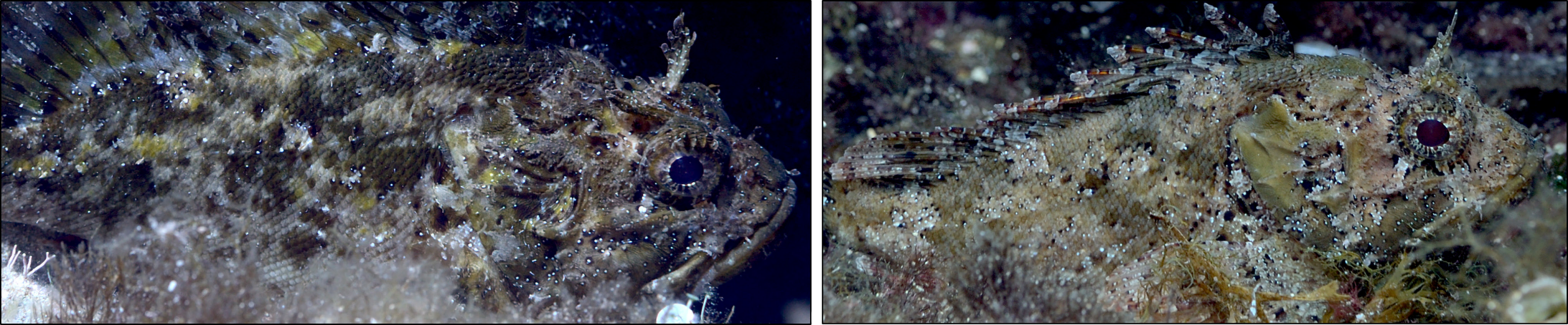
Two *Scorpaena porcus* individuals with different skin patterns. Photos by MS.

Scorpionfishes are sit‐and‐wait predators that show various camouflage strategies (John et al., 2023; Santon et al., 2018). Studying their ability to dynamically change body colouration can help to understand how they improve camouflage and therefore potentially increase predation success. A previous study has shown that the two species *Scorpaena maderensis* and *Scorpaena porcus* can rapidly adapt to background colour (John et al., 2023). Field observations indicate that *S. porcus* individuals vary in their skin pattern (Figure 1, personal observations by LJ). While those might be individual differences only, scorpionfishes' ability to change body colour raises the possibility that they can also adapt their pattern in response to specific background features.

Therefore, we tested whether both scorpionfish species adjust their body pattern in response to background pattern granularity (i.e. the patch size within the pattern) to increase background pattern matching. After an acclimation phase on a uniform grey background, we placed individuals on three experimental backgrounds of different granularity but similar average luminance and contrast estimated using the spectral sensitivities of scorpionfish (Govardovskii & Zueva, 1988; John et al., 2023; Schweikert et al., 2018). The *medium granularity* background was designed based on the average scorpionfish body patch size observed in a previous study (John et al., 2023), the *fine granularity* and *coarse granularity* backgrounds instead had a smaller and larger patch size. We documented scorpionfish body pattern after 1 min on each background using calibrated image analysis and compared whether their pattern differed depending on the background granularity. We expected that fish would change their pattern granularity depending on background granularity. In particular, we expected fish to show smaller patch size on the fine granularity background and larger patch size on the coarse granularity background when compared to their patch size on the medium granularity background. We decided to include fish pattern contrast into our analysis because we also suspected that scorpionfish could increase pattern contrast on the high‐contrasting experimental backgrounds, regardless of background granularity, when compared to the uniform acclimation background. We used different image analysis approaches to compare scorpionfish pattern metrics calculated from the visual perspective of a potential prey fish, the triplefin *Tripterygion delaisi*.

## 2 MATERIALS AND METHODS

### 2.1 Experimental animals

Experiments were carried out in the Station de Recherches Sous‐marines et Océanographiques (STARESO), Corsica, France in June and July 2022 and followed the general procedure and setup used by John et al. (2023). Madeira rockfish *Scorpaena maderensis* and the black scorpionfish *Scorpaena porcus* were caught with hand nets while SCUBA diving under the station's general sampling permit. We followed the EU animal welfare legislation's directive (Directive 2010/63/EU) to ensure that our research was not likely to cause pain, suffering, distress or lasting harm equivalent to, or higher than, that caused by the introduction of a needle in accordance with good veterinary practice. Fish were kept in shaded outside flow‐through seawater tanks (210 × 120 × 50 cm/1200 L) exposed to the natural light cycle. Both species are ambush predators that sit motionless on various natural hard substrates and feed on small fish and invertebrates (Louisy, 2002). Both species can adjust body colouration to the background in less than a minute (John et al., 2023). Observations in the field show a high pattern variability between individuals, yet it remains unclear whether scorpionfish can adjust skin pattern to that of the background (Figure 1).

### 2.2 Experimental setup

**FIGURE 2:**
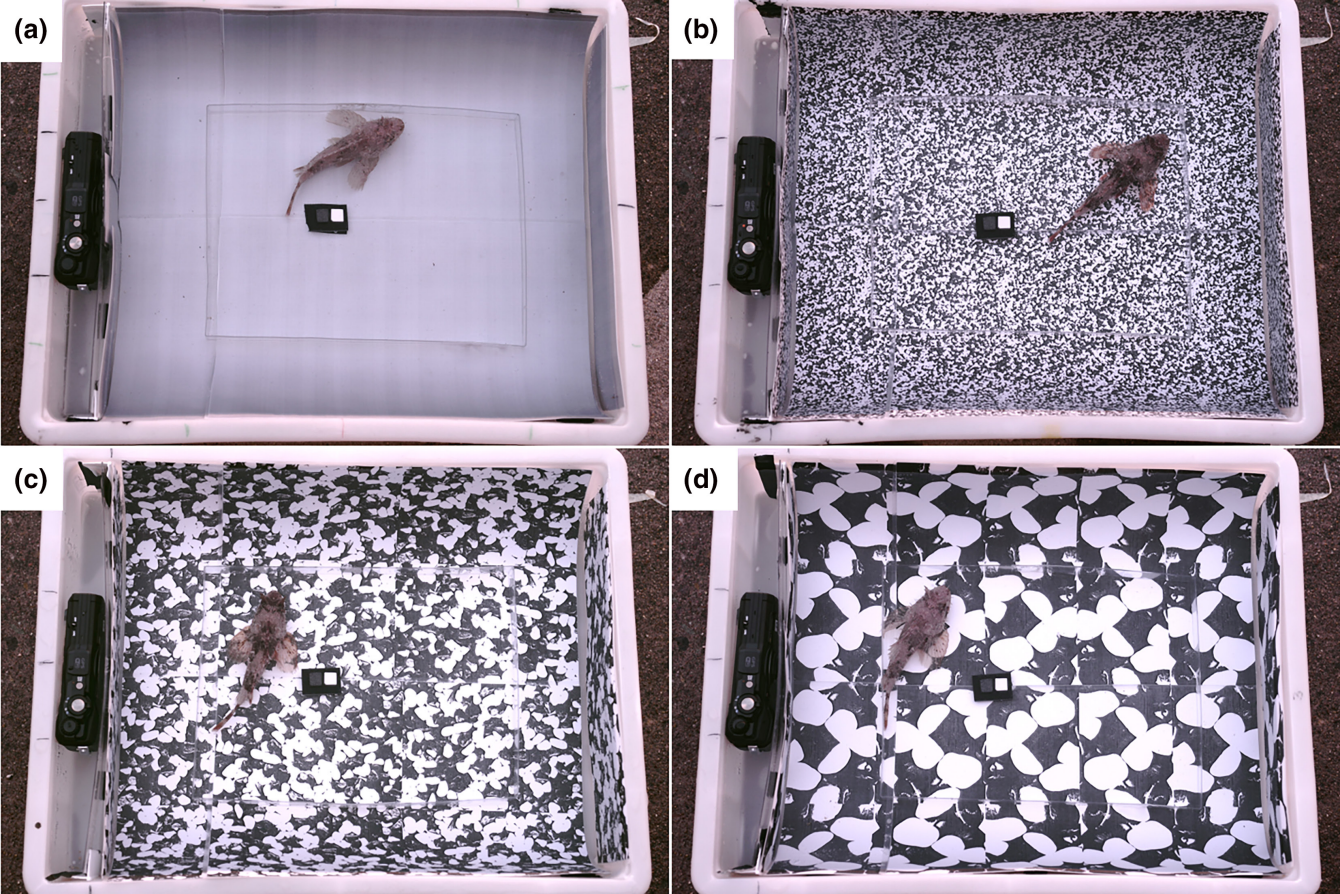
Exemplary top view photos in the experimental setup with the same *Scorpaena porcus* individual on (a) the acclimation background and the three experimental backgrounds with (b) fine, (c) medium and (d) coarse granularity. Body area of this individual was 6.5 cm^2^. The left side of each tray has a small compartment with a camera for side view photos. Fish are kept in the centre of the tray with a transparent plastic frame (best visible in a).

To elicit changes in body pattern, fish were alternately placed in three polyethylene trays (40 × 30 × 9 cm), each with a background of different pattern granularity (Figure 2). An identical tray was used for initial acclimation but had a uniform grey background. Backgrounds were printed on underwater paper (no. 3487; Avery Zweckform GmbH, Germany) with a laser printer (Kyocera ECOSYS P7240cdn KX, Kyocera, Japan) and then laminated with matte laminating pouches (125 micron, no. S‐PP525‐22, PRT GmbH, Switzerland). The three experimental backgrounds were black‐and‐white patterns of different granularity (fine, medium, coarse). Patterned backgrounds were created by taking photos of sand, gravel and small pebbles of different sizes. We used ImageJ (version 1.53o; Schneider et al., 2012) to convert the photos into masks showing 50% black and 50% white, to keep the contrast and average luminance perceived by the fish similar. The medium granularity roughly matched the average stripe size of scorpionfish estimated from a previous study (average grain size = 0.4 cm^2^; John et al., 2023). The other two granularities were distinctly smaller (average grain size = 0.1 cm^2^) and larger (average grain size = 1 cm^2^). To create the uniform acclimation background, we took standardised photos of the three experimental and acclimation backgrounds of different grey levels in the setup, and calculated their average luminance using scorpionfish spectral sensitivity (Govardovskii & Zueva, 1988; John et al., 2023; Schweikert et al., 2018). The acclimation background's grey level chosen was the one closest to the average luminance of the experimental backgrounds.

Trials took place outside the station, in a shaded area under the open sky. A small transparent plastic frame of 24 × 18 cm and 2 cm height was placed in the centre of the tray to prevent fish from hiding in the corners or edges of the tray (Figure 2). Top view photos of fish were taken using a calibrated Nikon D4 DLSR camera (NIKON CORPORATION, Tokyo, Japan, Nikkor 60 mm macro lens, RAW format, ISO and aperture fixed) positioned on a tripod at a 20° angle, and a ~100 cm distance from the tray. Each tray contained two centrally placed PTFE diffuse grey standards (12% and 72% grey, Berghof Fluoroplastic Technology GmbH, Germany) and a scale bar. Each tray was also equipped with an Olympus Tough TG‐6 camera (Olympus Europa SE & Co. KG, Hamburg, Germany, RAW format, ISO and aperture fixed) placed in a 3 cm wide compartment on the side of the tray (Figure 2). The camera was completely hidden during the trials, and only a small window for the lens was opened at the end of each trial to take a side view photo of the fish. We used a picture of a Mini ColourChecker Card (X‐Rite Inc., Grand Rapids, MI, USA) to calibrate the Olympus camera in the experimental setup under the same light conditions as in the experiments (Troscianko & Stevens, 2015; van den Berg et al., 2020).

### 2.3 Experimental procedure

We tested 21 *S. maderensis* and 30 *S. porcus*. Body size of the two species was similar on average (body area in top view for *S. maderensis*: 6.3 ± 2.0 cm^2^ (mean ± SD), and for *S. porcus*: 6.4 ± 2.3 cm^2^). Each individual was alternately placed on each experimental background. Trays were filled with fresh seawater before each trial. At the start of the experiment, each fish was first placed in the acclimation tray, and then on the three experimental backgrounds. We chose the uniform background as acclimation to obtain a reference image for each individual on a non‐patterned (uniform) background, and to acclimate the fish to the luminance of the experimental backgrounds before starting the trials. A fish was photographed from the top 1 min after being transferred in the tray (minute 1) and after 5 min (minute 5). Then, the transparent frame was removed and a piece of PVC tube with 12% and 72% grey standards oriented sideways was inserted in the tray, opposite to the side with the Olympus camera compartment. The fish was gently moved until it settled next to the standards. Then, the small window in the camera compartment was opened to take a side view photo. The fish was then placed in the next tray and the procedure was repeated for the other backgrounds. For transferring fish between backgrounds, we used hand nets. The six possible background orders (for the three experimental backgrounds) were randomised and fully balanced across all individuals of each species. All individuals were used only once and then returned to the field.

### 2.4 Image analysis

#### 2.4.1 Granularity analysis

**FIGURE 3:**
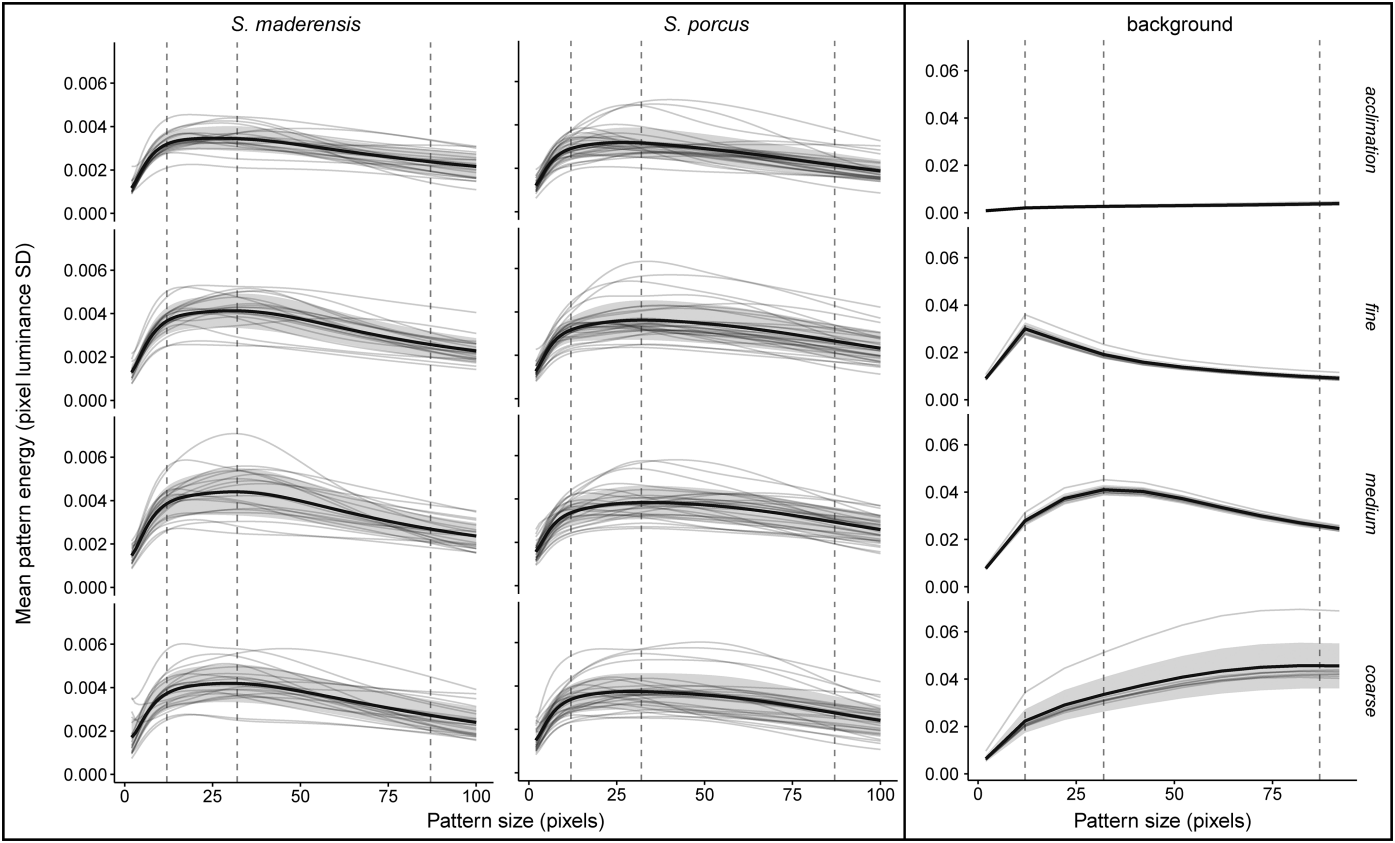
Pattern energy spectra (pattern energy for each pattern size bin) of the acclimation (uniform) and the three experimental backgrounds (right block), and of fish pattern for *Scorpaena maderensis* and *S. porcus* on each background (left block). Dashed vertical lines indicate the dominant marking size of the experimental background pattern (fine: *x* = 12, medium: *x* = 32, coarse: *x* = 87). The grey lines are spectra for each individual. The black lines indicate mean pattern energy over all individuals with standard deviation as the grey shaded area. Note the difference in pattern energy (range of y‐axis) between fish and backgrounds. Pattern energy is defined as the standard deviation of the luminance channel's cone catches of the filtered pixels (see Section 2).

**FIGURE A1:**
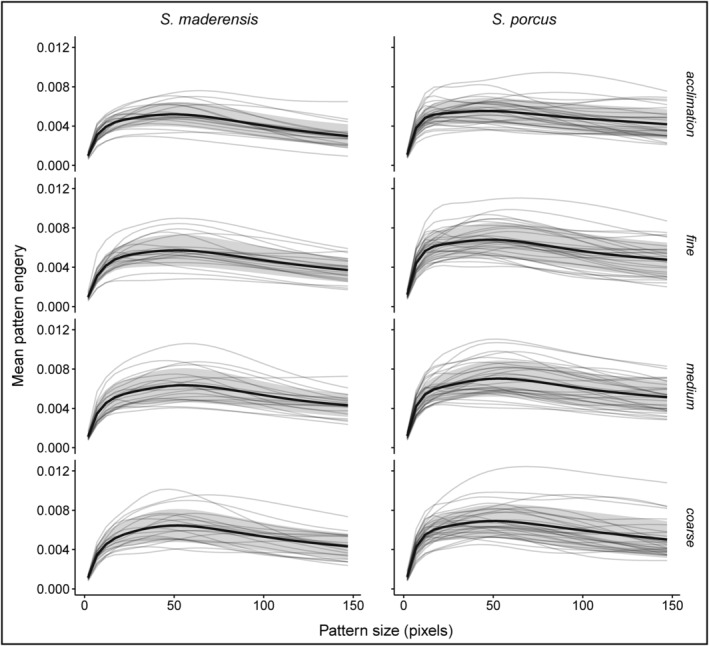
Pattern energy spectra (pattern energy for each pattern size bin) of *Scorpaena maderensis* (*n* = 20) and *S. porcus* (*n* = 30) on the acclimation (uniform) and the three experimental backgrounds based on the side view photos. The grey lines are spectra for each individual. The black lines indicate mean pattern energy over all individuals with standard deviation as the grey shaded area. Pattern energy is defined as the standard deviation of the luminance channel's cone catches of the filtered pixels (see Section 2).

To analyse fish body pattern, we used the multispectral image calibration and analysis (MICA) Toolbox plugin (version 2.2.2; Troscianko & Stevens, 2015) for ImageJ (version 1.54d). Images were normalised with the 12% and 72% grey standards and converted into 32‐bit multispectral images. For each image, we selected a region of interest (ROI) on the body of the fish. We always excluded the fins and paid attention to only select the part of the body that was illuminated at the same angle as the grey standards used to normalise the images. All photos were then batch‐processed using a custom‐written routine for MICA in ImageJ (John et al., 2023). First, body area for each fish was extracted as the number of pixels contained in the ROI ‘body’, to later calculate body area in cm^2^ using the scale in the photos. To exclude potential effects that fish body orientation in the tray could have on the pattern analysis, all top view photos were rotated in such a way that all fish were oriented in the same way. Then, images were converted to cone catches using a cone‐catch model that was computed using the spectral sensitivity of the camera and of a modelled observer, and the D65 spectrum as illuminant. We used D65 as illuminant because fish adjusted to backgrounds under this spectrum. We modelled the vision of the yellow black‐faced blenny *Tripterygion delaisi*, a common prey species of scorpionfish in the Mediterranean Sea (Santon et al., 2021), following previous studies (Bitton et al., 2017; John et al., 2023; Santon et al., 2020). *T. delaisi* has single cones with average peak sensitivity at 468 nm, and double cones with average sensitivity peaking at 517 and 530 nm (Bitton et al., 2017). We assumed a Weber fraction of 0.05 for the most abundant cones (Champ et al., 2016; Olsson et al., 2018), and estimated it to 0.1 for the short wavelength cones based on cone abundance ratios (from shortest to longest wavelength photoreceptor) of 1:4:4 (Fritsch et al., 2017). We defined the luminance channel as the average cone catches of the two longer wavelength‐sensitive cones, as fish likely perceive achromatic (luminance) contrasts through this channel (Lythgoe, 1979). The routine further processed the images to adjust for *T. delaisi* foveal spatial acuity of 7 cycles per degree (Fritsch et al., 2017; Santon et al., 2019), for a viewing distance of 20 cm (a relevant viewing distance in nature (Santon et al., 2021)), by using the Gaussian Acuity Control and the Receptor Noise Limited (RNL) Ranked Filter functions of the MICA toolbox (van den Berg et al., 2020). We then ran a granularity (pattern energy) analysis on the fish body using the ‘Pattern Colour & Luminance Measurements’ function of the toolbox (Troscianko & Stevens, 2015). This function uses fast Fourier transformation to produce images on different spatial scales and measures their pattern energy, defined as the standard deviation of the luminance channel's cone catches of the filtered pixels. By comparing pattern energy at different spatial scales (granularity bands), a dominant (highest energy, i.e. most contrasting) marking size can be determined (Barbosa et al., 2008; Stoddard & Stevens, 2010) (Figure 3, granularity spectrum for the four backgrounds). We analysed 99 granularity bands ranging from 2 to 100 pixels in size (i.e. using 1‐pixel steps) for the top view photos and 30 granularity bands ranging from 2 to 150 pixels (i.e. using 5‐pixel steps) for the side view photos. Analyses were stopped after 100 and 150 pixels because wider bin sizes exceeded the maximum fish and background patch size. Granularity bands differed between top and side view photos because the two cameras used had a different resolution, so analyses of top and side view photos can also not directly be compared (resolution of RNL rank filtered images: top view = 83 pixels per cm, side view = 80 pixels per cm). We visually inspected the granularity spectra derived from the side view photos and did not see any difference in fish pattern depending on the background (Figure A1). Because this was similar to the results derived from the top view photos, we focused on the top view for further analyses. To get the granularity spectra of the experimental backgrounds, we randomly chose eight top view photos per background type from our dataset and selected a 10 × 20 cm background patch in each image as an ROI ‘background’. This large sampling area was to ensure that we would capture the granularity of each background type. The photos were processed as described above. We analysed 15 granularity bands ranging from 2 to 150 pixels (i.e. using 10‐pixel steps) for the background samples. We reduced the number of granularity bands for the backgrounds to reduce computation time for the large samples and because the 15 bands seem to give a high enough resolution.

#### 2.4.2 QCPA analysis

The granularity analysis is widely used to assess dominant marking size in animal pattern research (Pérez‐Rodríguez et al., 2017). However, we decided to consider an additional approach to investigate fish pattern in more detail. We used RNL Clustering on the RNL rank‐filtered images to apply the colour adjacency analysis (CAA) from the Quantitative Colour Pattern Analysis (QCPA) (van den Berg et al., 2020). CAA creates clusters of pixels of the same colour and luminance within a pattern, based on a given perception threshold. We used the average size of these clusters as an additional measure of pattern granularity. While from the granularity analysis, we can extract the size of the most contrasting patches (dominant marking size), the CAA gives average patch size regardless of contrast (all contrasts above our given perception threshold). The comparison of these two metrics therefore allows us to understand whether fish change patch size overall (CAA) or specifically the dominant marking size (granularity analysis). We further used the local edge intensity analysis (LEIA) on the RNL rank‐filtered images to compare the mean luminance contrast value across edges within the fish body to test whether pattern contrast changed, irrespective of patch size (van den Berg et al., 2020). Chromatic contrasts were not analysed because, from *T. delaisi* perspective, there were almost no perceivable chromatic contrasts within the fish body pattern. For both analyses, we used a perception threshold of one just noticeable difference (JND) for *T. delaisi* vision. We ran the same analysis on the images with the background samples (see above).

### 2.5 Statistical analysis

We implemented generalised linear mixed models with the glmmTMB R‐package (Brooks et al., 2017) following a custom‐written guided linear modelling R‐routine (Santon et al., 2023). Model assessment followed the guidance of Santon et al. (2023), focusing on the inspection of the distribution of randomised quantile residuals, computed with the R‐package DHARMa (Hartig, 2022), within and among factor predictor levels that were included or not in the models, and performed posterior predictive checks to assess model dispersion and overall model fit. Models were initially implemented using the most appropriate family distribution for the nature of the response variable.

**FIGURE A2:**
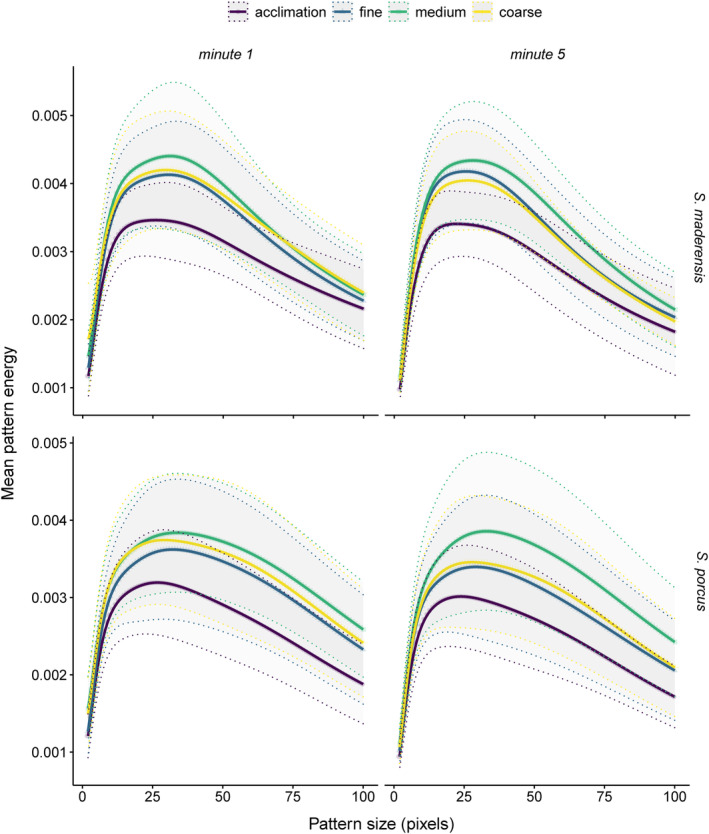
Pattern energy spectra (pattern energy for each pattern size bin) of *Scorpaena maderensis* (*n* = 21) and *S. porcus* (*n* = 30) on the acclimation (uniform) and the three experimental backgrounds, split by the timepoint at which the measurement was taken (after 1 or 5 min). The thick lines indicate mean pattern energy, the dotted lines indicate its standard deviation. Pattern energy is defined as the standard deviation of the luminance channel's cone catches of the filtered pixels (see Section 2).

Data analysed originated from 51 individuals (21 *S. maderensis* and 30 *S. porcus*). We only analysed observations after 1 min of exposure to the backgrounds because our previous study showed that scorpionfish change colour in less than 1 min (John et al., 2023), and because the granularity analysis spectra comparing measurements after 1 and 5 min were similar (Figure A2). To compare fish patterns on the different experimental backgrounds, we implemented generalised linear mixed models using a Gamma distribution (link = log) for the response variables *dominant marking size* (granularity analysis), *patch size* (CAA) and *pattern contrast* (LEIA), and specified *background* (fine, medium, coarse), scorpionfish *species* (*S. maderensis, S. porcus*), and their interaction as fixed effects in each model. *Fish ID* was used as a random intercept to account for the repeated measurements of each fish. We added random slopes over a specific predictor when effect sizes' direction substantially varied among fish (Korner‐Nievergelt et al., 2015). We therefore only included a random slope over *background* in the *dominant marking size* model.

To further investigate whether fish pattern contrast changed between the acclimation and the first experimental background, we created a subset of the data that only included observations for the acclimation and the first experimental background each fish was tested on. We implemented a generalised linear mixed model using a Gamma distribution (link = log) for the response variable *pattern contrast* (LEIA), with *event* (acclimation, first experimental background) and *first background type* (fine, medium, coarse) as main fixed effects. *First background type* was a variable created to group observations of the acclimation with the first experimental background type and included to compare whether a change in contrast differed between the experimental backgrounds. *Fish ID* was included as a random intercept.

We report *R* ^2^‐values as a measure of fit for each model and report both the marginal R^2^ (variance explained by fixed effects only) and the conditional *R* ^2^ (variance explained by entire model) (Nakagawa & Schielzeth, 2013) (Tables 1, 2), using the *r2* function of the performance package (Lüdecke et al., 2021). For graphical displays of the results, our figures present model predicted means and their 95% compatibility intervals calculated from the posterior distributions of fitted values obtained from 10,000 sets of model parameters (Brooks et al., 2017). We further used the emmeans package (Lenth, 2023) to compute pairwise contrasts expressed as ratios between factor levels and their 95% compatibility intervals for all combinations of factor predictors of interest (Tables, 1, 2). Effect size strength increases with increasing deviation of differences from one, and the robustness of the result increases with decreasing degree of overlap of the 95% compatibility intervals (CIs) with one.

## 3 RESULTS

### 3.1 Change in pattern granularity

#### 3.1.1 Dominant marking size (granularity analysis)

**FIGURE 4:**
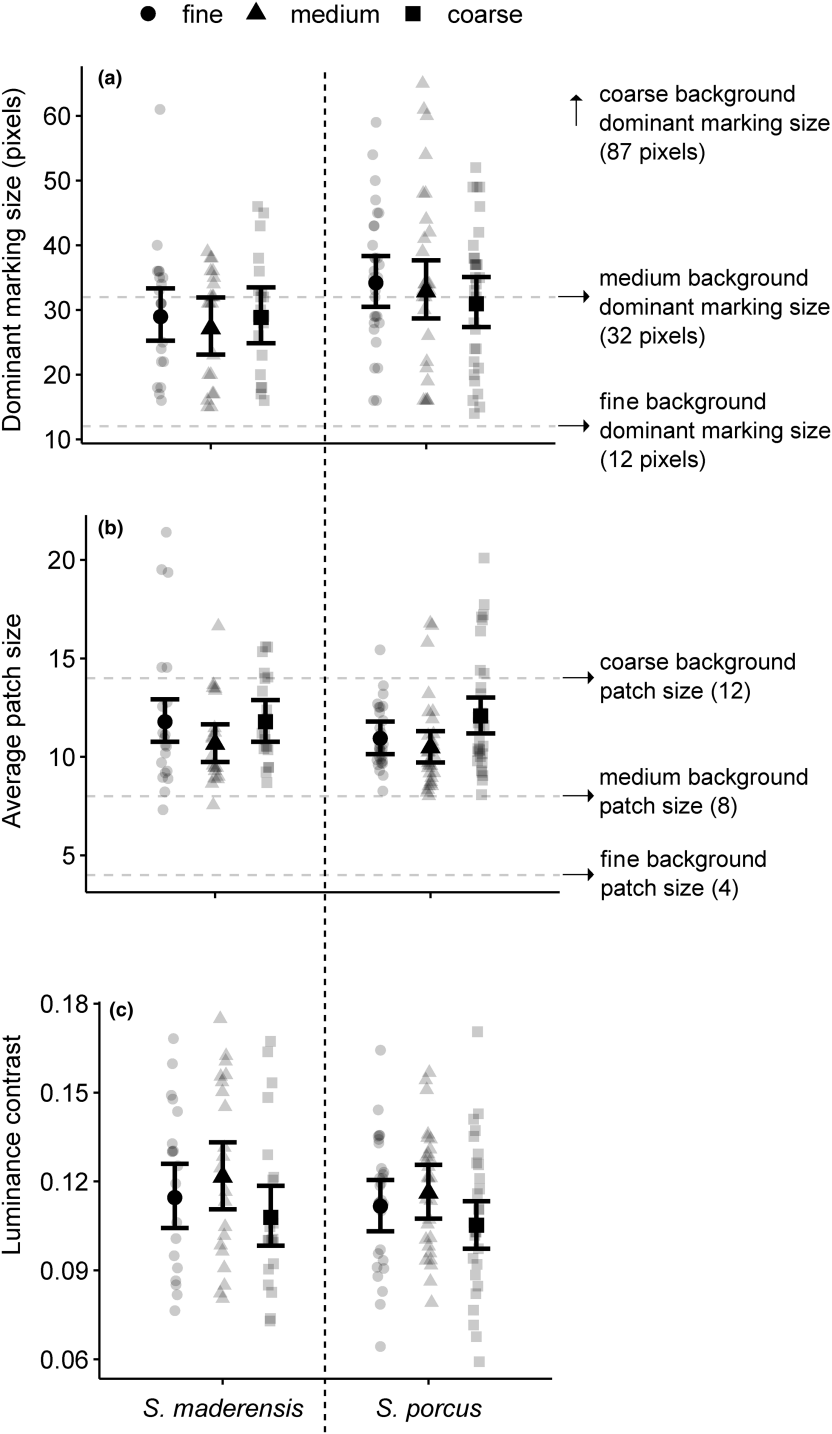
Fish pattern metrics depending on background granularity. (a) Dominant marking size is the spatial scale (measured in pixels) showing the highest contrast per individual fish, based on the granularity analysis. Dashed horizontal lines indicate the dominant marking size of the experimental backgrounds (fine: *y* = 12, medium: *y* = 32, coarse: *y* = 87). (b) Average patch size is the average size of clusters derived from the RNL clustered image of the fish body in the CAA. Dashed horizontal lines indicate the average patch size of the experimental background pattern (fine: *y* = 4, medium: *y* = 8, coarse: *y* = 14). (c) Luminance contrast is given as the mean contrast value of edges based on the LEIA. Points represent observations for each individual fish (*N* = 21 *Scorpaena maderensis*, *N* = 30 *S. porcus*). Markers with vertical bars represent predicted medians and 95% compatibility intervals (CIs) derived from 10,000 simulations of the posterior distribution of model parameters. The strength of the difference between two groups increases with decreasing degree of overlap of their 95% CIs.

From inspecting the granularity spectra, we cannot see changes in dominant marking size when fish were placed on backgrounds of different granularity (Figures 3 and 4a; Table 1A). This becomes particularly evident when looking at how the spectra instead differed between backgrounds (Figure 3). On average, fish show a constant dominant marking size similar to that of the medium granularity background (Figure 4a). However, fish have a relatively heterogenous pattern granularity. While the mean curves peak at around 32 pixels, pattern energy remains high between ~20 and 40 pixels (Figure 3). For very regular patterns, a steeper peak around dominant marking size would be expected. Dominant marking size of *S. maderensis* is similar to that of *S. porcus* (dominant marking size between species ratio averaged over *background*: 0.87, 95% CI 0.75–1.01). Variance of dominant marking size was higher for *S. porcus* (*σ* ^2^ = 97.98) than for *S. maderensis* (*σ* ^2^ = 77.45), while their body sizes were comparable (see Section 2).

**TABLE 1:** Pairwise contrasts of fish pattern (A) dominant marking size, (B) average patch size and (C) luminance contrast expressed as the response ratio between all combinations of background for both scorpionfish species.

	*Scorpaena maderensis*			*Scorpaena porcus*		
	Response ratio	Lower CIs	Upper CIs	Response ratio	Lower CIs	Upper CIs
(A) Dominant marking size ($R_{\text{cond}}^{2}$ = .637, $R_{\text{marg}}^{2}$ = .050)						
Fine – medium	1.07	0.90	1.27	1.04	0.90	1.20
Fine – coarse	1.00	0.82	1.22	1.10	0.94	1.30
Medium – coarse	0.94	0.81	1.09	1.06	0.94	1.20
(B) Average patch size ($R_{\text{cond}}^{2}$ = .511, $R_{\text{marg}}^{2}$ = .066)						
Fine – medium	**1.11**	**1.01**	**1.22**	1.04	0.97	1.13
Fine – coarse	1.00	0.91	1.10	**0.91**	**0.84**	**0.98**
Medium – coarse	**0.90**	**0.82**	**0.99**	**0.87**	**0.80**	**0.94**
(C) Luminance contrast ($R_{\text{cond}}^{2}$ = .813 $R_{\text{marg}}^{2}$ = .045)						
Fine – medium	**0.94**	**0.89**	**0.99**	0.96	0.91	1.01
Fine – coarse	1.06	1.00	1.13	**1.06**	**1.01**	**1.11**
Medium – coarse	**1.13**	**1.06**	**1.20**	**1.10**	**1.05**	**1.16**

*Note*: Effect size is proportional to the deviation of ratios from one, and the robustness of the result increases with decreasing degree of overlap of the 95% compatibility intervals (CIs) with one. Response ratios with CIs excluding one are highlighted in bold. *N* = 21 for *S. maderensis* and *N* = 30 for *S. porcus*.

#### 3.1.2 Average patch size (CAA)

Both species show differences in average patch size depending on experimental backgrounds. *S. maderensis* shows a smaller average patch size on the medium, compared to the fine and coarse background (Figure 4b, Table 1B). *S. porcus* shows a larger average patch size on the coarse, compared to the medium and fine background (Figure 4b, Table 1B). Patch size of *S. maderensis* is similar to that of *S. porcus* (patch size between species ratio averaged over *background*: 1.02, 95% CI 0.93 to 1.13).

### 3.2 Change in pattern luminance contrast (LEIA)

#### 3.2.1 Comparison between experimental backgrounds

Both species show a lower pattern luminance contrast on the coarse, compared to the medium granularity background (Figure 4c, Table 1C). Pattern contrast of *S. maderensis* is similar to that of *S. porcus* (pattern contrast between species ratio averaged over *background*: 1.03, 95% CI 0.92 to 1.16).

#### 3.2.2 Comparison between acclimation and first experimental background

**FIGURE 5:**
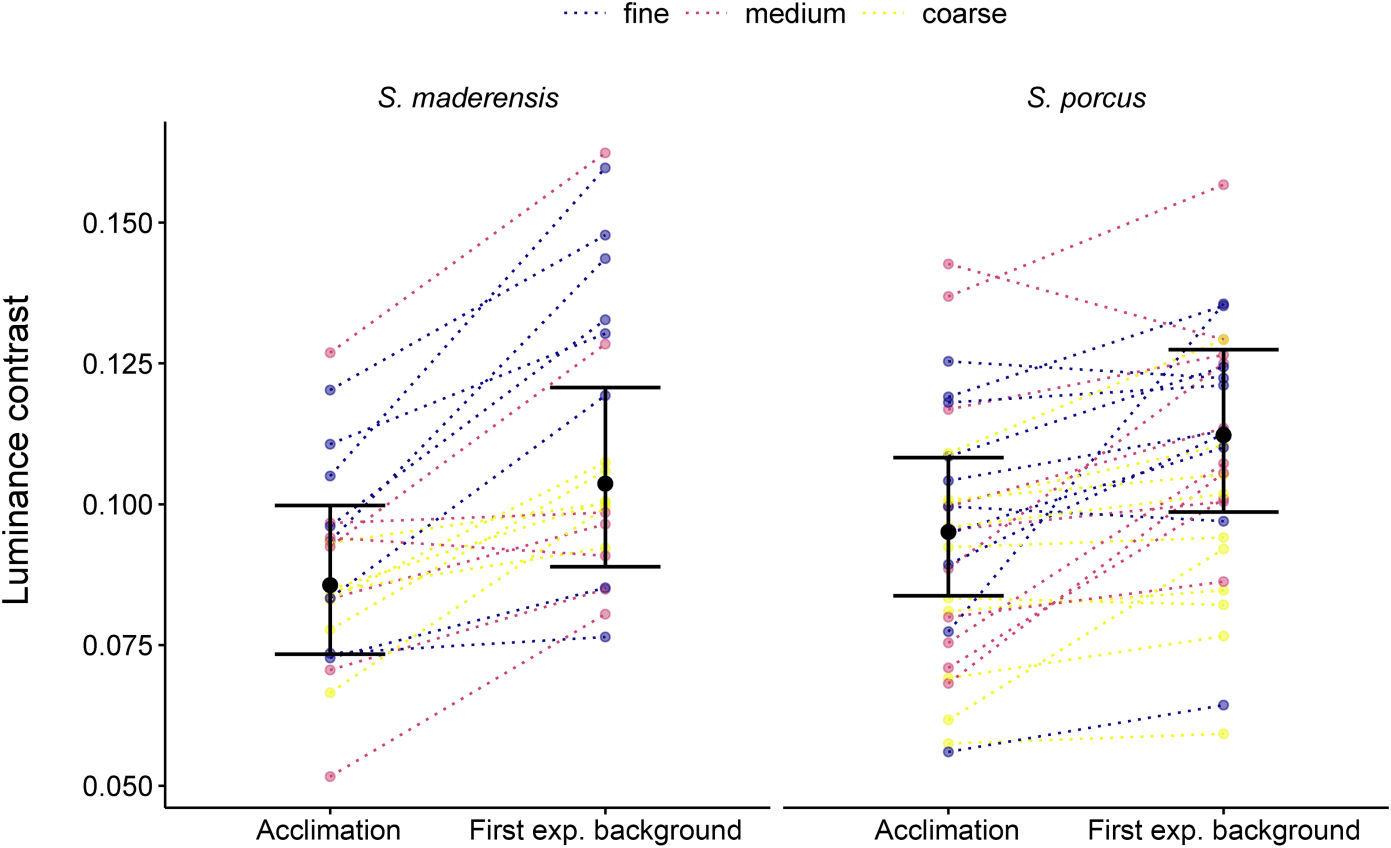
Fish change their pattern contrast between acclimation and the first experimental background. Contrast is given as the mean contrast value of edges based on the LEIA. The horizontal lines connect data points of an individual, colours indicate the background type used in the first experimental background. Points represent observations for each individual fish (*N* = 21 *Scorpaena maderensis*, *N* = 30 *S. porcus*). Markers with vertical bars represent predicted medians and 95% compatibility intervals (CIs) derived from 10,000 simulations of the posterior distribution of model parameters. The strength of the difference between two groups increases with decreasing degree of overlap of their 95% CIs.

Fish increased the contrast of their pattern when moved from the acclimation to the first experimental background, regardless of its granularity (Figure 5, Table 2).

**TABLE 2:** Pairwise contrasts of fish pattern luminance contrast (mean contrast of edges in LEIA) expressed as the response ratio between the acclimation and the first experimental background for both scorpionfish species, either pooling all measurements in the first experimental background regardless of background type, or split by background.

	Response ratio	Lower CIs	Upper CIs
*Scorpaena maderensis*			
Acclimation – first experimental background (pooled)	**0.80**	**0.75**	**0.85**
Acclimation – fine	**0.77**	**0.69**	**0.85**
Acclimation – medium	**0.83**	**0.74**	**0.92**
Acclimation – coarse	**0.81**	**0.72**	**0.91**
*Scorpaena porcus*			
Acclimation – first experimental background (pooled)	**0.87**	**0.83**	**0.92**
Acclimation – fine	**0.87**	**0.80**	**0.95**
Acclimation – medium	**0.83**	**0.76**	**0.91**
Acclimation – coarse	**0.91**	**0.83**	**0.99**

*Note*: Effect size is proportional to the deviation of ratios from one, and the robustness of the result increases with decreasing degree of overlap of the 95% compatibility intervals (CIs) with one. Response ratios with CIs excluding one are highlighted in bold. *N* = 21 for *S. maderensis* and *N* = 30 for *S. porcus*. $R_{\text{cond}}^{2}$ = .820, $R_{\text{marg}}^{2}$ = .254.

## 4 DISCUSSION

We investigated whether the two scorpionfish species *Scorpaena maderensis* and *S. porcus* change their pattern depending on the granularity of their visual background. Fish changed their average patch size and pattern contrast. However, dominant marking size, the most contrasting component of the pattern (Barbosa et al., 2008; Stoddard & Stevens, 2010), was not modulated on different granularity backgrounds. This was in contrast to other camouflaged benthic fishes such as different species of flounder, which can adapt their body pattern dominant marking size flexibly to different substrate granularities (Akkaynak et al., 2017; Ramachandran et al., 1996). Possibly, scorpionfish did not change in our experiment because the right cues to induce pattern change, such as specific pattern components, or even tactile or olfactory cues, were missing (Stevens & Ruxton, 2019). However, there might well be morphological or physiological restraints that prevent scorpionfish from modulating pattern elements, such as the inability to regulate the chromatophores of different skin patches independently. Similar to other fishes such as the rock pool goby *Gobius paganellus* (Smithers et al., 2017), or the flatfishes *Paralichthys lethostigma* and *Pseudopleuronectes americanus* (Saidel, 1977), scorpionfish seem to have one dominant body pattern with a given patch size, which can be modulated by adjusting the contrast between patches.

The Colour Adjacency Analysis revealed small changes in average patch size depending on background granularity. This indicates that fish changed their pattern in response to the background, but without modulating their dominant, most contrasting marking size. *S. maderensis* have the smallest average patch size on the medium granularity background, and *S. porcus* have a smaller patch size on both fine and medium granularity backgrounds. However, the average patch size remains substantially larger than that of the medium granularity background. Therefore, it is unclear how these small observed changes may affect the fishes' camouflage. It is indeed more plausible that changes in average patch size might only be a by‐product of the changes in pattern contrast that we observed. As fish modulate contrast within their pattern, perceived size of some patches may vary due to some edges blending in or becoming more apparent. Pattern contrast of both species in the Local Edge Intensity Analysis was highest on the medium granularity background. Possibly, fish increased pattern contrast especially on the medium granularity background because this is closest to their own dominant marking size. Increasing pattern contrast on a similar background could improve background pattern matching by intensifying the pattern through the increasing contrast. Furthermore, all fish had a substantially lower pattern contrast in the acclimation uniform background compared to the fine, medium and coarse experimental ones. While this could be a result of differences in pattern granularity, it is likely that the difference in contrast induced this change, as the acclimation was the only background without contrast. On backgrounds with high‐contrast patterns, fish might increase their pattern contrast regardless of background granularity to improve disruption by displaying maximum disruptive contrast (Stevens & Merilaita, 2009b). We suggest that both contrast and granularity may impact the pattern regulation of the fish. It is known that cuttlefish use both pattern size and pattern contrast as cues to adjust their body pattern and that backgrounds with higher contrast elicit body patterns with higher contrast (Barbosa et al., 2008).

An individual fish pattern is relatively heterogenous in terms of dominant marking size and average patch size, meaning fish have patches of different sizes and not a very regular pattern. This could function as a generalist body pattern that works well on multiple backgrounds, reducing the need to modulate body pattern (Briolat et al., 2021; Merilaita et al., 1999), a strategy known from animals found on highly complex and heterogenous backgrounds (Hughes et al., 2019; Nokelainen et al., 2019). Moreover, scorpionfish could show further adaptations that improve their camouflage and reduce the need to adjust pattern to different backgrounds, such as an active background selection (Stevens & Ruxton, 2019). While we overall observed similar results for both scorpionfish species, there seems to be a higher individual variation of dominant marking size in *S. porcus* compared to *S. maderensis*, and this cannot be explained by a systematic variation in body size. High individual variation in pattern could benefit camouflage by disrupting the search image of prey or preventing search image formation (Bond & Kamil, 2002; Stevens et al., 2014; Surmacki et al., 2013). Individual pattern variation can also be favoured by living in a very heterogeneous habitat (Merilaita et al., 1999), and it is possible that the species differ in their microhabitat use with *S. porcus* living in more complex microhabitats or having a more generalist habitat use. An assessment of scorpionfish colouration and behaviour in their natural environment could help to understand the importance of skin pattern for their camouflage and consequently, prey capture success.

## AUTHOR CONTRIBUTIONS

**Leonie John:** Conceptualization (equal); data curation (lead); formal analysis (lead); investigation (lead); methodology (lead); visualization (lead); writing – original draft (lead); writing – review and editing (equal). **Matteo Santon:** Conceptualization (equal); methodology (supporting); writing – review and editing (equal). **Nico K. Michiels:** Conceptualization (equal); writing – review and editing (equal).

## CONFLICT OF INTEREST STATEMENT

The authors declare that they have no competing interests.

## Data Availability

All data collected and analysed in the study will be available on Figshare, https://doi.org/10.6084/m9.figshare.24560323.
